# Immune-related adverse events associated with PD-1 and PD-L1 inhibitors for nonsmall cell lung cancer: Protocol for a systematic review and meta-analysis

**DOI:** 10.1097/MD.0000000000008407

**Published:** 2017-11-03

**Authors:** Xiaoying Sun, Raheleh Roudi, Shangya Chen, Bin Fan, Hong Jin Li, Min Zhou, Xin Li, Bin Li

**Affiliations:** a Department of Dermatology, Yueyang Hospital of Integrated Traditional Chinese and Western Medicine, Shanghai University of Traditional Chinese Medicine; b Institute of Dermatology, Shanghai Academy of Traditional Chinese Medicine, Shanghai, China; c Oncopathology Research Center, Iran University of Medical Sciences, Tehran, Iran; d Department of Toxicology, Shandong Academy of Occupational Health and Occupational Medicine, Shandong academy of Medical Science, Jinan, Shandong, China.

**Keywords:** immune-related adverse events, inhibitors, meta-analysis, nonsmall cell lung cancer, programmed cell death protein-1, programmed death-ligand 1, systematic review

## Abstract

**Introduction:**

Nonsmall cell lung cancer accounts for approximately 80% of all lung cancers, and approximately 75% of cases are diagnosed in the middle and late stages of disease. Unfortunately, limited treatment does not improve the prognosis of advanced disease. Monoclonal antibodies targeting programmed cell death protein-1 (PD-1) and programmed death-ligand 1 (PD-L1) represent a new treatment paradigm for nonsmall cell lung cancer. Nevertheless, the immune-related adverse events (irAEs) associated with PD-1 and PD-L1 inhibitors are unique, and early recognition and treatment of these events are essential.

**Methods and Analysis:**

A systematic literature search will be performed using the EMBASE, MEDLINE, and Cochrane databases to identify relevant articles published in any language. Randomized clinical trials, case series, and case reports of PD-1 and PD-L1 inhibitors in the treatment of nonsmall cell lung cancer will be included. All meta-analyses will be performed using RevMan software. The quality of the studies will be evaluated using the guidelines listed in the Cochrane Handbook. If the necessary data are available, then subgroup analyses will be performed for high-, median-, and low-dose cohorts. The Preferred Reporting Items for Systematic Reviews and Meta-Analyses statements will be followed until the findings of the systematic review and meta-analysis are reported.

**Conclusions:**

This will be the first systematic review and meta-analysis to describe previously reported irAEs related to PD-1 and PD-L1 inhibitors in the treatment of nonsmall cell lung cancer.

## 1 Introduction

Lung cancer remains a leading cause of cancer-related mortality,^[1]^ with nonsmall cell lung cancer (NSCLC) accounting for 80% to 85% of all cases of lung cancer.^[2]^ Based on its pathology, NSCLC can be further subdivided into adenocarcinoma, squamous cell carcinoma, and large cell carcinoma. Compared with small cell lung cancer, the growth of NSCLC cells is slower, and the diffusion occurs relatively late. If diagnosed early, the disease generally has a good prognosis, with surgical resection providing a clinical cure. However, NSCLC is diagnosed as locally advanced or metastatic disease in 70% of patients,^[3]^ leading to an extremely poor 5-year survival rate of 1% to 5% among patients with stage IV NSCLC, a figure that has not changed over the past 25 years.^[4]^

Although first-line systemic treatment for NSCLC consists of surgery followed by cytotoxic chemotherapy and/or radiation therapy, increasing recognition of the critical roles of the immune system in the development and progression of cancer has spurred the development of numerous targeted therapies with significant potential to improve overall survival (OS) in specific NSCLC populations.^[5]^ Currently, it is well established that tumors suppress antitumor immune responses via numerous mechanisms including the production of inhibitory cytokines, recruitment of immunosuppressive immune cells, and upregulation of coinhibitory receptors known as immune checkpoints.^[6]^ In normal physiological environments, the immune checkpoints contribute to inhibiting immune responses against self-antigens to prevent excessive autoimmunity. However, these immune checkpoints are upregulated in many cancers, resulting in cancer-associated immune suppression and immune evasion.^[7]^ PD-1 binds to PD-L1 and decreases effector cytokine production and cytolytic activity, preventing the elimination of cancer.^[8]^ This discovery prompted the development of different monoclonal antibodies targeting either PD-1 or PD-L1 as new treatments for advanced NSCLC.

PD-1 is a type I transmembrane protein receptor expressed on the surface of numerous immune cells such as macrophages, dendritic cells, T-cells, B-cells, and natural killer cells.^[7,9]^ PD-L1 and PD-L2 both act on PD-1.^[10,11]^ However, the main function of T-cell regulation within the tumor microenvironment depends on PD-L1, which is expressed on the same cells that express PD-1. In addition, some cancer cells also express PD-L1,^[12]^ and approximately 60% of NSCLCs are PD-L1–positive.^[13]^ Blockade of the PD-1 pathway is believed to increase the anti-tumor immune function of T-cells, enhance natural killer cell activity, and promote antibody production in PD-1–positive B cells.^[7,9]^

Currently, pembrolizumab and nivolumab, which target PD-1 receptors, have been approved for the first- and second-line treatment of NSCLC, respectively.^[5,14,15]^ Other PD-1/PD-L1 inhibitors are in various stages of clinical development. The induction of a tolerance break against the tumor via PD-1/PD-L1 blockade can lead to immune dysregulation, namely immune-related adverse events (irAEs), which clinically manifest as symptoms including dermatologic, gastrointestinal, hepatic, endocrine, and pulmonary events.^[16]^ In addition to these unique AEs, treatment-related adverse events (trAEs) include fatigue, anorexia, nausea, and diarrhoea.^[5,14,17–21]^

As these treatment-related symptoms affect patients’ quality of life, sufficient knowledge, and appropriate management of these events are important.^[22,23]^ Our study will assess the incidence and nature of irAEs in the oncologic treatment of NSCLC using available anti-PD-1 or anti-PD-L1 antibodies through a systematic review and meta-analysis of the qualifying literature.

## 2 Objective

A systematic review and meta-analysis will be performed to clarify the profile of irAEs associated with PD-1 and PD-L1 inhibitors in the treatment of NSCLCs in a systematized manner and summarize the differences in the adverse effects associated with different inhibitors compared with conventional chemotherapy and radiotherapy, including whether these events are dose-dependent.

### 2.1 Primary objective

- To describe the profile of irAEs associated with PD-1 and PD-L1 inhibitors in the treatment of NSCLCs in a systematized manner.

### 2.2 Secondary objectives

- To compare the irAEs associated with PD-1 and PD-L1 inhibitors with those associated with conventional chemotherapy and radiotherapy.
- To compare the trAEs associated with PD-1 and PD-L1 inhibitors with those associated with conventional chemotherapy and radiotherapy.
- To determine whether the severity and frequency of irAEs associated with PD-1 and PD-L1 inhibitors are dose-dependent.
- To assess whether the profile of irAEs differs among different PD-1 and PD-L1 inhibitors.

## 3 Methods

### 3.1 Eligibility criteria

#### 3.1.1 Types of participants

The included participants will be adults who were diagnosed with NSCLC and treated with anti-PD-1 or anti-PD-L1 antibodies. Previous oncologic therapy will not preclude study inclusion, but anti-PD-1 or anti-PD-L1 antibodies must be administered separately without combination therapy. Eligible comparison treatments will be chemotherapy, radiation therapy, targeted therapy, immunity therapy, and multiple combination therapies. There will be no restrictions regarding sex, race/ethnicity, education, and economic status.

#### 3.1.2 Types of studies

This study will include randomized controlled trials (RCTs), case series, and case reports published in peer-reviewed journals without language or time restrictions. Cohort studies, case-control studies, cross-sectional studies, postmarketing vaccine surveillance studies, and other nonrandomized studies will be excluded.

#### 3.1.3 Exclusion criteria

Review articles, non-RCTs, or RCTs combining anti-PD-1 antibodies or anti-PD-L1 antibodies with other treatment modalities will be excluded.

### 3.2 Outcomes

The incidence evaluation will be based on the number of irAEs and their grades [1–5, recorded according to Version 2, 3, or 4 of the Common Terminology Criteria for Adverse Events (CTCAE) of the National Cancer Institute]. Grades ≥ 3 will be considered high-grade.

#### 3.2.1 Primary outcomes

- The incidences of irAEs and trAEs associated with PD-1 and PD-L1 inhibitors in the treatment of NSCLC.
- The incidences of irAEs and trAEs associated with conventional chemotherapy and radiotherapy in the treatment of NSCLC.
- The incidences of organ-specific adverse events (AEs), especially immune-related pneumonia, and deaths related to AEs associated with PD-1 and PD-L1 inhibitors in the treatment of NSCLCs.
- The incidences of organ-specific AEs, especially immune-related pneumonia, and deaths related to AEs associated with conventional chemotherapy and radiotherapy in the treatment of NSCLCs.

#### 3.2.2 Secondary outcomes

- Comparison of the incidence of irAEs and trAEs between PD-1 and PD-L1 inhibitors and conventional chemotherapy/radiotherapy.
- Comparison of the incidence of organ-specific AEs, especially immune-related pneumonia, and deaths related to AEs between PD-1 and PD-L1 inhibitors conventional chemotherapy/radiotherapy.

### 3.3 Information sources

A systematic literature search through December 2016 will be performed using EMBASE, MEDLINE via PubMed, and the Cochrane Central Register of Controlled Trials for relevant articles published in any language.

### 3.4 Search strategy

The relevant searching terms will match Medical Subject Heading terms, and the searches will be repeated immediately before the final analyses to identify additional studies for inclusion. An example of the PubMed search strategy is shown in Table 1.

**Table 1:**
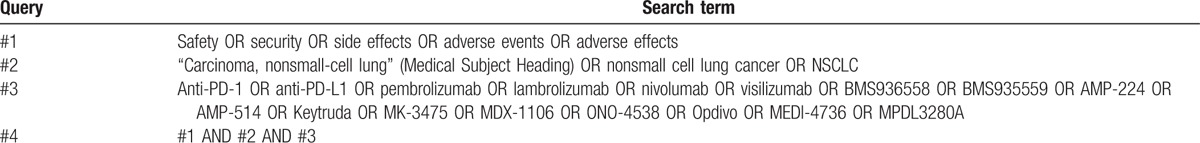
Search strategy for PubMed.

### 3.5 Study records

#### 3.5.1 Data management

An electronic parent folder will be created for this study. The folder will contain several subfolders to incorporate all records retrieved using the following criteria: database of retrieval, the full text of the included studies, the results of risk of bias assessments and subgroup analyses, and full systematic review manuscript drafts. The aforementioned task will be the responsibility of the first author (XYS) in consultation with another author (SYC). The parent folder will be backed up on 2 author's mobile hard drives.

#### 3.5.2 Selection process

Two authors (XYS and SYC) will screen the search outputs using titles and abstracts to assess whether they meet the inclusion criteria as defined by the protocol and then read the full text of all potentially eligible studies independently for further discrimination of eligible studies. The full text will be obtained through databases or by contacting the corresponding author of the article that will be in the charge of another 2 authors (BF and RR). Discrepancies regarding study inclusion between the 2 authors will be resolved via consensus with the assistance of the senior author.

#### 3.5.3 Data collection process and data items

Data will be extracted from the included studies by 2 authors (XYS and SYC) independently and recorded on a predesigned extraction list. The following data will be captured in the systematic review and meta-analysis:

1. Study characteristics: author, clinical trial information, design, and enrolment size.
2. Characteristics of monoclonal antibodies: type and dose.
3. Diagnostic methods: Version 2, 3 or 4 of the Common Terminology Criteria for Adverse Events of the National Cancer Institute
4. IrAEs/trAEs: type, frequency of any grade, frequency of severe grade, and the median time to onset.
5. Frequency of organ-specific AEs, especially immune-related pneumonia, and the incidence of death related to irAEs.

### 3.6 Assessment of risk of bias

The Cochrane Collaboration's ‘risk of bias’ tool will be used to assess the risk of bias and evaluate the quality of articles included in the systematic review and meta-analysis,^[24]^ and to address selection bias, performance bias, detection bias, attrition bias, reporting bias, and other biases. Two authors will independently conduct this quality assessment, with agreement reached via consensus.

### 3.7 Data synthesis

All meta-analyses will be performed using RevMan 5.2 software. Statistical heterogeneity between the selected studies will be assessed using the *χ*^*2*^ test and I-squared statistic. Subgroup analyses will be performed to explore potential causes of heterogeneity. The relative risk will be calculated for dichotomous data with 95% confidence intervals for all analyses.

#### 3.7.1 Subgroup analysis

If sufficient data can be obtained, then subgroup analyses will be conducted for high-, median-, and low-dose cohorts. Other variables that will be considered for subgroup analysis include the type (anti-PD-1 vs anti-PD-L1) and brand of antibody drugs.

#### 3.7.2 Sensitivity analysis

Sensitivity analysis will be performed to confirm whether the results are robust and credible by excluding highly biased studies.

### 3.8 Reporting of the review

The preferred reporting items for systematic reviews and meta-analyses (PRISMA) statement will be followed until the findings of the systematic review and meta-analysis are reported. The identification and selection of studies for inclusion will be summarized using flow diagrams. Characteristics of the studies and the incidence of global adverse events associated with anti-PD-1 and anti-PD-L1 therapies will be presented in tables. The quality assessment and summary of other adverse events will be described in the text.

## 4 Discussion

To our knowledge, this will be the first systematic review and meta-analysis reporting all published irAEs related to PD-1 and PD-L1 inhibitors in the treatment of NSCLC. Currently, the study of anti-PD-1 and anti-PD-L1 transitions from basic research to clinical research; however, safety concerns represent the primary limitation of the clinical application stage. Using all RCTs, case series, and case reports included in the pooled analysis, we will determine the incidences of all-grade and high-grade irAEs, which may highlight the high risk of irAEs associated with anti-PD-1 and anti-PD-L1 drugs in the treatment of NSCLC.

PD-1 and PD-L1 inhibitors are demanded because of the poor prognosis of NSCLC despite their potentially high risk of irAEs. Several antibodies have been approved for the second-line and higher treatment of NSCLC, and they are associated with significant improvements in progression-free survival and OS versus conventional chemotherapy ^[15]^; thus, security details need to be observed and monitored in the clinical practice. This systematic review will be conducted to clarify essential details associated with anti-PD-1 and anti-PD-L1 drugs in the treatment of NSCLC and the findings of the review may facilitate early prediction, comprehensive observation, and prompt management of irAEs in addition to better patient compliance.

We will report the review results according to PRISMA guidelines and search, screen, assess, and extract valuable data from several databases comprehensively and meticulously as previously mentioned. As trAEs may be reported instead of irAEs in studies of anti-PD-1 and anti-PD-L1 drugs in patients with NSCLC, it may be impossible to conduct the review as described in this article. This consideration will be addressed in the discussion of published studies, and the study results will be disseminated in peer-reviewed journals.

## References

[1] American Cancer Society. Cancer facts and Figures 2015. Atlanta: American Cancer Society; 2015.

[2] Sher T Dy GK Adjei AA Small cell lung cancer. Mayo Clin Proc 2008;83:355–67. 18316005 10.4065/83.3.355

[3] Molina JR Yang P Cassivi SD Non-small cell lung cancer: epidemiology, risk factors, treatment, and survivorship. Mayo Clin Proc 2008;83:584–94. 18452692 10.4065/83.5.584 PMC2718421

[4] Howlader N Noone AM Krapcho M SEER cancer statistics review 1975–2013. Bethesda: National Cancer Institute 2016.

[5] Borghaei H Paz-Ares L Spigel DR Nivolumab versus docetaxel in advanced nonsquamous non-small-cell lung cancer. N Engl J Med 2015;373:1627–39. 26412456 10.1056/NEJMoa1507643 PMC5705936

[6] Drake CG Jaffee E Pardoll DM Mechanisms of immune evasion by tumors. Adv Immunol 2006;90:51–81. 16730261 10.1016/S0065-2776(06)90002-9

[7] Pardoll D The blockade of immune checkpoints in cancer immunotherapy. Nav Rev Cancer 2012;12:252–64. 10.1038/nrc3239 PMC4856023 22437870

[8] Jiang Y Li Y Zhu B T-cell exhaustion in the tumor microenvironment. Cell Death Dis 2015;6:e1792. 26086965 10.1038/cddis.2015.162 PMC4669840

[9] La-Beck N Jean G Huynh C Immune checkpoint inhibitors: new insights and current place in cancer therapy. Pharmacotherapy 2015;35:963–76. 26497482 10.1002/phar.1643

[10] Jing W Li M Zhang Y PD-1/PD-L1 blockades in non-small-cell lung cancer therapy. Onco Targets Ther 2016;9:489–502. 26889087 10.2147/OTT.S94993 PMC4741366

[11] Keir ME Butte MJ Freeman GJ PD-1 and its ligands in tolerance and immunity. Annu Rev Immunol 2008;26:677–704. 18173375 10.1146/annurev.immunol.26.021607.090331 PMC10637733

[12] Sundar R Cho BC Brahmer JR Nivolumab in NSCLC: latest evidence and clinical potential. Ther Adv Med Oncol 2015;7:85–96. 25755681 10.1177/1758834014567470 PMC4346216

[13] Sheppard KA Fitz LJ Lee JM PD-1 inhibits T-cell receptor induced phosphorylation of the ZAP70/CD3ζ signalosome and downstream signaling to PKCθ. FEBS Lett 2004;574:37–41. 15358536 10.1016/j.febslet.2004.07.083

[14] Brahmer J Reckamp K Baas P Nivolumab versus docetaxel in advanced squamous-cell non-cell lung cancer. N Engl J Med 2015;373:123–35. 26028407 10.1056/NEJMoa1504627 PMC4681400

[15] Le AD Alzghari SK Jean GW Update on targeted therapies for advanced non-small cell lung cancer: nivolumabin context. Ther Clin Risk Manag 2017;13:223–36. 28260909 10.2147/TCRM.S104343 PMC5328134

[16] Michot JM Bigenwald C Champiat S Immune-related adverse events with immune checkpoint blockade: a comprehensive review. Eur J Cancer 2016;54:139–48. 26765102 10.1016/j.ejca.2015.11.016

[17] Robert C Long GV Brady B Nivolumab in previously untreated melanoma without BRAF mutation. N Engl J Med 2015;372:320–30. 25399552 10.1056/NEJMoa1412082

[18] Weber JS D’Angelo SP Minor D Nivolumab versus chemotherapy in patients with advanced melanoma who progressed after anti-CTLA-4 treatment (CheckMate 037): a randomised, controlled, open-label, phase 3 trial. Lancet Oncol 2015;16:375–84. 25795410 10.1016/S1470-2045(15)70076-8

[19] Ribas A Puzanov I Dummer R Pembrolizumab versus investigator-choice chemotherapy for ipilimumab-refractory melanoma (KEYNOTE-002): A randomised, controlled, phase 2 trial. Lancet Oncol 2015;16:908–18. 26115796 10.1016/S1470-2045(15)00083-2 PMC9004487

[20] Herbst RS Baas P Kim DW Pembrolizumab versus docetaxel for previously treated, PD-L1- positive, advanced non-small-cell lung cancer (KEYNOTE-010): a randomised controlled trial. Lancet 2016;387:1540–50. 26712084 10.1016/S0140-6736(15)01281-7

[21] Fehrenbacher L Spira A Ballinger M Atezolizumab versus docetaxel for patients with previously treated non-small-cell lung cancer (POPLAR): a multicentre, open-label, phase 2 randomised controlled trial. Lancet 2016;387:1837–46. 26970723 10.1016/S0140-6736(16)00587-0

[22] Bacon CG Giovannucci E Testa M The association of treatment-related symptoms with quality-of-life outcomes for localized prostate carcinoma patients. Cancer 2002;94:862–71. 11857323 10.1002/cncr.10248

[23] Butler L Bacon M Carey M Determining the relationship between toxicity and quality of life in an ovarian cancer chemotherapy clinical trial. J Clin Oncol 2004;22:2461–8. 15197209 10.1200/JCO.2004.01.106

[24] Higgins JPT Altman DG Gotzsche PC The Cochrane Collaboration's tool for assessing risk of bias in randomized trials. BMJ 2011;43:d5928. 10.1136/bmj.d5928 PMC3196245 22008217

